# Supplementary material for: Development and Validation of the Pain and State of Health Inventory (PHI): Application for the Perioperative Setting
Source: J Clin Med. 2021 May 3;10(9):1965. doi: 10.3390/jcm10091965 (PMC8124984; doi:10.3390/jcm10091965)
Supplement: Supplementary file 1 [file jcm-10-01965-s001.zip › jcm-1160600-supplementary.pdf]

PHI

I Pain

1. Do you currently have pain?
   ☐ Yes ☐ No (***if no**, please go to subsection II Emotional states, p. 2*)
   ***If yes,** please answer the following questions about your pain:*

2. Where do you have pain?

1.	Mouth/ Face/ Head	☐
2.	Neck and throat region	☐
3.	Shoulder/ Arm/ Hand	☐
4.	Chest area	☐
5.	Abdominal area	☐
6.	Back area	☐
7.	Hip/ Leg/ Foot	☐
8.	Pelvic area	☐
9.	Genitals/ anus	☐
10.	Joints	☐
11.	Entire body	☐

If you have pain **in more than one area**, please answer the following questions in relation to your **pain that is currently most severe.**

3. On the following scale, please mark the number that best represents the intensity of your pain.
   Example: Your pain is of medium intensity
   (0) (1) (2) (3) (4) ~~(5)~~ X (6) (7) (8) (9) (10)
   No pain — Worst pain imaginable

3a. How severe is your pain?
At rest…
(0) (1) (2) (3) (4) (5) (6) (7) (8) (9) (10)
No pain — Worst pain imaginable

3b. How severe is your pain?
With physical activity (e.g. walking, sitting, picking up something from the floor)…
(0) (1) (2) (3) (4) (5) (6) (7) (8) (9) (10)
No pain — Worst pain imaginable

4. Since when do you have this pain? *Please mark the applicable answer category*

☐	☐	☐	☐	☐
For hours	For days	For weeks	Up to half a year	For have a year or longer

5. How often do you have the pain? *Please mark the applicable answer category*

☐	☐	☐	☐	☐	☐
Constantly	Several times a day	Once a day	Every couple of days	Every few weeks	rarer

6. Is this pain related to the upcoming surgery?
   Yes ☐ No ☐

© Klinger, R., Krug F., Stuhlreyer, J., 2021

II <u>Emotional states</u>

The next questions are about how you are feeling. For each statement, please mark the number that corresponds best to the intensity of emotional state.

You do not feel anxious at all

(0)	(1)	(2)	(3)	(4)	(5)	(6)	(7)	(8)	(9)	(10)
Not anxious at all										Very anxious

You feel very anxious

(0)	(1)	(2)	(3)	(4)	(5)	(6)	(7)	(8)	(9)	(10)
Not anxious at all										Very anxious

How do you feel the last days and today?

a) I feel **sad**:

(0)	(1)	(2)	(3)	(4)	(5)	(6)	(7)	(8)	(9)	(10)
Not sad at all										Very sad

b) I feel **anxious:**

(0)	(1)	(2)	(3)	(4)	(5)	(6)	(7)	(8)	(9)	(10)
Not anxious at all										Very anxious

c) I am **tired:**

(0)	(1)	(2)	(3)	(4)	(5)	(6)	(7)	(8)	(9)	(10)
Not tired at all										Very tired

d) I feel **numb:**

(0)	(1)	(2)	(3)	(4)	(5)	(6)	(7)	(8)	(9)	(10)
Not numb at all										Very numb

e) I feel **weak:**

(0)	(1)	(2)	(3)	(4)	(5)	(6)	(7)	(8)	(9)	(10)
Not weak at all										Very weak

f) I feel **irritated:**

(0)	(1)	(2)	(3)	(4)	(5)	(6)	(7)	(8)	(9)	(10)
Not irritated at all										Very irritated

g) My **mood** is overall:

(0)	(1)	(2)	(3)	(4)	(5)	(6)	(7)	(8)	(9)	(10)
Good, without impairment										Bad, severe impairment

© Klinger, R., Krug F., Stuhlreyer, J., 2021

III <u>General state of health</u>

For this question, please indicate how you would rate your overall health. Please mark the category that applies best to you.

How are you? My general state of health is:

1.	Very good	☐
2.	Good	☐
3.	Rather good	☐
4.	Moderate	☐
5.	Rather bad	☐
6.	Bad	☐
7.	Very bad	☐
8.	I do not know	☐

© Klinger, R., Krug F., Stuhlreyer, J., 2021

PHI

I <u>Pain</u>

1. Do you currently have pain?
   ☐ Yes ☐ No (***if no**, please go to subsection II Emotional states, p. 2*)
   ***If yes,** please answer the following questions about your pain:*

2. Where do you have pain?

1.	Mouth/ Face/ Head	☐
2.	Neck and throat region	☐
3.	Shoulder/ Arm/ Hand	☐
4.	Chest area	☐
5.	Abdominal area	☐
6.	Back area	☐
7.	Hip/ Leg/ Foot	☐
8.	Pelvic area	☐
9.	Genitals/ anus	☐
10.	Joints	☐
11.	Entire body	☐

If you have pain **in more than one area**, please answer the following questions in relation to the pain which is related to the surgery**.**

3. On the following scale, please mark the number that best represents the intensity of your pain.
   Example: Your pain is of medium intensity
   (0) (1) (2) (3) (4) X(5) (6) (7) (8) (9) (10)
   No pain Worst pain imaginable

3a. How severe is your pain related to your surgery?
At rest…
(0) (1) (2) (3) (4) (5) (6) (7) (8) (9) (10)
No pain Worst pain imaginable

3b. How severe is your pain related to your surgery?
With physical activity (e.g. walking, sitting, and picking up something from the floor)…
(0) (1) (2) (3) (4) (5) (6) (7) (8) (9) (10)
No pain Worst pain imaginable

© Klinger, R., Krug F., Stuhlreyer, J., 2021

II <u>Emotional states</u>

The next questions are about how you are feeling. For each statement, please mark the degree that corresponds best to the intensity of emotional state.

You do not feel anxious at all

☒(0)	(1)	(2)	(3)	(4)	(5)	(6)	(7)	(8)	(9)	(10)

Not anxious at all — Very anxious

You feel very anxious

(0)	(1)	(2)	(3)	(4)	(5)	(6)	(7)	(8)	(9)	☒(10)

Not anxious at all — Very anxious

How did you feel the last days and today?

a) I feel **sad**:

(0)	(1)	(2)	(3)	(4)	(5)	(6)	(7)	(8)	(9)	(10)

Not sad at all — Very sad

b) I feel **anxious:**

(0)	(1)	(2)	(3)	(4)	(5)	(6)	(7)	(8)	(9)	(10)

Not anxious at all — Very anxious

c) I am **tired:**

(0)	(1)	(2)	(3)	(4)	(5)	(6)	(7)	(8)	(9)	(10)

Not tired at all — Very tired

d) I feel **numb:**

(0)	(1)	(2)	(3)	(4)	(5)	(6)	(7)	(8)	(9)	(10)

Not numb at all — Very numb

e) I feel **weak:**

(0)	(1)	(2)	(3)	(4)	(5)	(6)	(7)	(8)	(9)	(10)

Not weak at all — Very weak

f) I feel **irritated:**

(0)	(1)	(2)	(3)	(4)	(5)	(6)	(7)	(8)	(9)	(10)

Not irritated at all — Very irritated

g) My **mood** is overall:

(0)	(1)	(2)	(3)	(4)	(5)	(6)	(7)	(8)	(9)	(10)

Good, without impairment — Bad, severe impairment

© Klinger, R., Krug F., Stuhlreyer, J., 2021

III General state of health

For this question, please indicate how you would rate your overall health. Please mark the category that applies best to you.

How are you? My general state of health is:

1.	Very good	☐
2.	Good	☐
3.	Rather good	☐
4.	Moderate	☐
5.	Rather bad	☐
6.	Bad	☐
7.	Very bad	☐
8.	I do not know	☐

IV Somatic parameters

Mobility

Please indicate how much exercise you did today. Please mark the appropriate category.

How much did you move today?

1.	I was only in bed	☐
2.	I had physiotherapy in bed	☐
3.	I sat down in bed	☐
4.	I went to the bathroom with help of others	☐
5.	I walked around with the help of physiotherapist/ nurse	☐
6.	I went to the bathroom without help	☐
7.	I walked around with the help of others (e.g. visitors)	☐
8.	I walked around without help of others	☐

Do you think, you could have done more exercise?

Yes, I could have done more exercise	☐
I am content with my level of exercise	☐
No, I was overstrained with my level of exercise	☐

Diet

Please indicate what you ate/drunk today. Please mark the appropriate category.

1.	No food/ drink	☐
2.	Limited tea/ water	☐
3.	Unlimited tea/ water	☐
4.	Soup/ yoghurt	☐
5.	Mashed food	☐
6.	Light to normal diet (including diabetic food)	☐

© Klinger, R., Krug F., Stuhlreyer, J., 2021

Do you think, you could have tolerated more/other nourishments?

Yes, I would have tolerated more/ other nourishments	☐
I tolerated everything I ate/drunk	☐
No, I did not tolerate the received nourishments	☐

© Klinger, R., Krug F., Stuhlreyer, J., 2021

PHI – External assessment for nursing care, physiotherapists, and physicians

Preoperatively

Nursing Care

<u>General state of health</u>

For this question, please indicate how you would rate the overall health of the patient. Please mark the category that applies best.

How is the patient? The general state of health of the patient is:

1.	Very good	☐
2.	Good	☐
3.	Rather good	☐
4.	Moderate	☐
5.	Rather bad	☐
6.	Bad	☐
7.	Very bad	☐
8.	I do not know	☐

Physician

<u>Lung function</u>: FEV_1 ___________________ FVC ______________________

<u>Analgesics</u>: No ☐ Yes ☐ Which? _________________________________

How much? ______________________________

<u>Health condition assessment:</u>

Please indicate how you would currently assess the patient's health condition generally.

1.	Very good health condition	☐
2.	Good health condition	☐
3.	Rather good health condition	☐
4.	Moderate health condition	☐
5.	Rather bad health condition	☐
6.	Bad health condition	☐
7.	Very bad health condition	☐
8.	I do not know	☐

© Klinger, R., Krug F., Stuhlreyer, J., 2021

Postoperatively

Nursing Care

General state of health

For this question, please indicate how you would rate the overall health of the patient. Please mark the category that applies best.

How is the patient? The general state of health of the patient is:

1.	Very good	☐
2.	Good	☐
3.	Rather good	☐
4.	Moderate	☐
5.	Rather bad	☐
6.	Bad	☐
7.	Very bad	☐
8.	I do not know	☐

Somatic parameters

For this question, please indicate what the patient ate/drunk today. Please mark the appropriate category.

1.	No food/ drink	☐
2.	Limited tea/ water	☐
3.	Unlimited tea/ water	☐
4.	Soup/ yoghurt	☐
5.	Mashed food	☐
6.	Light to normal diet (including diabetic food)	☐

Physiotherapists

Somatic parameters

Please indicate how much exercise the patient did today. Please mark the appropriate category.

How much did the move today?

1.	S/he was only in bed	☐
2.	S/he had physiotherapy in bed	☐
3.	S/he sat down in bed	☐
4.	S/he went to the bathroom with help of others	☐
5.	S/he walked around with the help of physiotherapist/ nurse	☐
6.	S/he went to the bathroom without help	☐
7.	S/he walked around with the help of others (e.g. visitors)	☐
8.	S/he walked around without help of others	☐

© Klinger, R., Krug F., Stuhlreyer, J., 2021

Physician

Lung function: FEV_1 ___________________ FVC ______________________

Analgesics: No ☐ Yes ☐ Which? _________________________________

How much? ______________________________

Received PCA: No ☐ Yes ☐ How much? ______________________________

Status evaluation:

Please indicate how you would currently assess the patient's health condition generally.

1.	Very good health condition	☐
2.	Good health condition	☐
3.	Rather good health condition	☐
4.	Moderate health condition	☐
5.	Rather bad health condition	☐
6.	Bad health condition	☐
7.	Very bad health condition	☐
8.	I do not know	☐

© Klinger, R., Krug F., Stuhlreyer, J., 2021
